# Wild meat hunting and use by sedentarised Baka Pygmies in southeastern Cameroon

**DOI:** 10.7717/peerj.9906

**Published:** 2020-09-17

**Authors:** Eva Avila Martin, Guillermo Ros Brull, Stephan M. Funk, Luca Luiselli, Robert Okale, Julia E. Fa

**Affiliations:** 1 Zerca y Lejos ONGD, Madrid, Spain; 2 Nature Heritage, Jersey, Channel Islands; 3 Institute for Development, Ecology, Conservation and Cooperation, Rome, Italy; 4 Department of Applied and Environmental Biology, Rivers State University of Science and Technology, Port Harcourt, Nigeria; 5 Department of Natural Sciences, School of Science and the Environment, Manchester Metropolitan University, Manchester, United Kingdom; 6 CIFOR Headquarters, Center for International Forestry Research (CIFOR), Bogor, Indonesia

**Keywords:** Bushmeat, Prey species composition, Harvest rates, Extraction rates, Trapping, Shot guns, Africa

## Abstract

As a result of sedentarisation many Baka Pygmies have changed their mobility patterns away from nomadic lifestyles to living in roadside villages. These settled groups are increasingly dependent on cultivated foods but still rely on forest resources. The level of dependence on hunting of wild animals for food and cash, as well as the hunting profiles of sedentarised Pygmy groups is little known. In this study we describe the use of wild meat in 10 Baka villages along the Djoum-Mintom road in southeastern Cameroon. From data collected from 1,946 hunting trips by 121 hunters, we show that most trips are of around 13 hours and a median of eight hours. A mean ± SD of 1.15 ± 1.11 animal carcasses are taken in a single trip; there was a positive correlation between duration of trips and carcasses. A total of 2,245 carcasses of 49 species of 24 animal families were taken in the study; species diversity was similar in all villages except one. Most hunted animals were mammals, with ungulates contributing the highest proportion. By species, just over half of the animal biomass extracted by all hunters in the studied villages was provided by four mammal species. Most animals were trapped (65.77% ± 16.63), followed by shot with guns (22.56% ± 17.72), other methods (8.69% ± 6.96) and with dogs (2.96% ± 4.49). A mean of 7,569.7 ± 6,103.4 kg yr^−1^ (2,080.8–19,351.4) were extracted per village, giving 75,697 kg yr^−1^ in total, which is equivalent to 123 UK dairy cattle. In all villages, 48.07% ± 17.58 of animals hunted were consumed by the hunter and his family, around 32.73% ± 12.55, were sold, followed by a lower percentage of carcasses partially sold and consumed (19.21% ± 17.02). Between 60% and 80% of carcasses belonged to the “least concern” category, followed by “near threatened”, “vulnerable” and, rarely “endangered”. The only endangered species hunted was the chimpanzee (*Pan troglodytes*). We suggest that hunting is a critical activity that provides a vital source of food for our study communities. Measured wild meat extraction levels are likely to be sustainable if hunter densities do not increase.

## Introduction

Wildlife is hunted in African forest and savanna regions as a source of meat and income, to control agricultural crop pests, reduce threats to livestock and human safety, and as trophies (Coad et al., 2018). However, there are concerns that the overexploitation of wildlife across sub-Saharan Africa will lead to the loss of an important source of dietary protein, micro-nutrients, and income for numerous rural poor (Bennett et al., 2007; Nasi, Taber & Van Vliet, 2011) and imperil the cultural identities of many local and traditional people for which hunting is part of their heritage and sense of self (Van Vliet & Mbazza, 2011). When hunter-gatherer groups are few and range across large landscapes that they defend as “their” exclusive territory, hunting of all wildlife species can be sustainable (Bennett et al., 2007). However, hunting can rapidly become unsustainable, as has happened in some groups in central Africa (e.g., Riddell, 2013), if they switch from being wild meat consumers to traders supplying local or distant markets (Inogwabini, 2014; Van Vliet et al., 2017).

Pygmy^1^ 1The term Pygmy is sometimes considered pejorative, though it is still widely employed in the literature (see Hewlett, 2014). Some authors suggest that its use is inexact since it a broad ”category” that does not capture the diversity of societies that make up the rainforest hunter-gatherers of the Congo Basin (Robillard & Bahuchet, 2012). However, a term that can replace Pygmy has not been universally agreed upon. The use of a self-applied term for ‘forest people’ (bisi ndima), such as BaYaka, as suggested by Lewis (2015), could be the solution. In this paper, we continue to use Pygmy or Pygmies to denote all Indigenous peoples in the Congo Basin. Despite this, this term has not been universally adopted e.g., Duda (2017) writes “I will mostly use it for reason of convenience, when discussing about cross-cultural researches and commonalities between groups”.peoples are distributed throughout the Congo basin in Africa. Most of the dozen or so ethno-linguistically distinct groups recognised by anthropologists (Bahuchet, 2014) live in rainforests as forest foragers and hunter-gatherers; two groups, the Bedzan (Medzan) of Cameroon and the Twa of Rwanda and Burundi, live in non-forest areas (Hewlett, 2014). All Pygmy groups share a hunter-gatherer lifestyle (and increasingly ‘former’ hunter-gatherer lifestyle). They have specific cultural practices and distinctive physical traits. In the Western Congo Basin, Pygmy groups include the Aka in Central African Republic (CAR) and Republic of Congo (RoC); Baka in RoC, Cameroon and Gabon; Bongo in Gabon and RoC; Luma, Mbendjele, Mikaya and Ngombe in RoC. In essence, the preeminent traditional way of life for these groups is associated with forest hunting and gathering even though some have taken up some form of agriculture. All meet their dietary protein needs almost exclusively from wildlife (Kelly, 2013; Hewlett, 2014; Duda, 2017).

The Baka in south–eastern Cameroon, formerly strict hunters-gatherers, differ in a number of attributes from neighbouring Bantu-speaking people. These traits (shared with other western Pygmies) are linked to a subsistence economy related to the exploitation of wild resources. Specifically, the Baka are characterized by high seasonal mobility associated to a specific forest habitat type, a mobility that is now maintained by shifting between settlement and forest camp-life (Dounias & Leclerc, 2006; Leclerc, 2012; Gallois, 2015), the use of a diversity of hunting techniques bound to their social structure and rituals (Joiris, 1998; Yasuoka, 2006; Yasuoka, 2014; Hayashi, 2008; Duda, Gallois & Reyes-Garcia, 2017) and a dietary dependence on resource gathering and collection (Bahuchet, 1992; Yasuoka, 2006; Gallois & Duda, 2016). The Baka also maintain a close association with sedentary Bantu-speaking swidden agriculturalists with whom they maintain complex social, economic, and symbolic relations (Joiris, 1998; Rupp, 2003).

The history of the Baka has been affected by the slave trade, by European colonialism and more recently by the clustering of populations along roads. Until the mid-twentieth century, the Baka were living in small groups of 30–40 individuals (Bahuchet, 1992) but the adoption of agriculture and their settlement along roads are arguably the two most important events during the 20th century. The latter phenomenon was the result of the implementation of “development assistance” programs by the State after independence, as early as 1960 (Leclerc, 2012); the assumption was that the settling down of nomadic peoples was a pathway to increased access to services. However, though these events are often depicted as imposed by colonial administration or missionaries, the adoption of agriculture and semi-sedentary lifestyle has been rather voluntary (Froment, 2014). The two events are the result of a more complex situation. The changing economic relations of the Baka with their neighbours might be at the origin of their settlement, but factors more related to internal social dynamics may also be involved (Pyhälä, 2012). In all cases, while drivers of Baka settlement are numerous, most of them indirectly relate to colonization, social restructuration, and resources trade. Leclerc (2012) also suggests that the decline in elephant populations, on which the Baka had been very dependent, may have contributed to the reorganization of Baka social units enabling the move to roadside villages.

Today, numerous Baka groups live in villages along the same road as neighbouring Bantu-speaking villagers/farmers. Since at least the 1950s (Althabe, 1965) resettled Baka have engaged in subsistence agriculture by opening their own plots (Kitanishi, 2003; Knight, 2003; Leclerc, 2012; Yasuoka, 2012). Sedentarised Baka groups, however, still depend on wild meat and other forest products for both their diet and income, often to different degrees (Hayashi, 2008; Yasuoka, 2014; Duda, 2017; Reyes-García et al., 2019; Gallois et al., 2020). Hunting studies of forest-living Pygmies throughout the Congo Basin show that mammals, especially ungulates and within this group duikers, are the main prey, and compared to non-Pygmy hunters they sell a very small proportion of animals hunted (Fa et al., 2016).

As a result of sedentarisation many Baka have changed their mobility patterns, which in themselves vary as related to life choices made at the individual household level (Althabe, 1965; Bahuchet, McKey & de Garine, 1991; Bailey, Bahuchet & Hewlett, 1992; Leclerc, 2012). Traditionally, Baka spatial organization would revolve around the various forest camps, which may change between seasons (Bahuchet, McKey & de Garine, 1991). During the last decades, this organization has reoriented away from truly nomadic living to one of life between village settlement and forest camps (Duda, 2017). This change in lifestyle has been associated with a marked decline in physical and mental health (Dounias & Froment, 2006; The Lancet, 2016). It has also had clear consequences on diets and food security, as shown by the better diets of Baka Pygmies living in isolated villages compared to those in villages closer to markets (Reyes-García et al., 2019). In another area, Hewlett (1987) similarly found that Aka Pygmy nutrition was considerably better in the forest because hunting for protein-rich game meat occurs regularly, while in the village camp they largely depended upon a starchy village diet of manioc and plantains. Research on some Baka groups in southeastern Cameroon indicate that, although their food consumption patterns had changed after social transition, supplementing their life in the village with time in forest camps reduced stress and helped them to maintain a better nutritional status (Hagino, Sato & Yamauchi, 2014).

In this paper, by employing community-based reporting schemes, we quantify the diversity of animals hunted and the techniques used by Baka communities living non-nomadic lives in south-eastern Cameroon. Specifically, we ask the following questions: What are the numbers and species diversity of animals hunted per hunter and village? How efficient are hunting trips? How much is used for own consumption and for sale? We discuss whether these extraction levels are likely to be sustainable and debate the importance of hunted wild meat for food security and income. We also compare our results with other hunting studies of Pygmy and non-Pygmy groups in the Congo Basin and assess the dissimilarities in numbers and biomass of animals extracted by each group.

## Materials and Methods

### Study area

**Figure 1:**
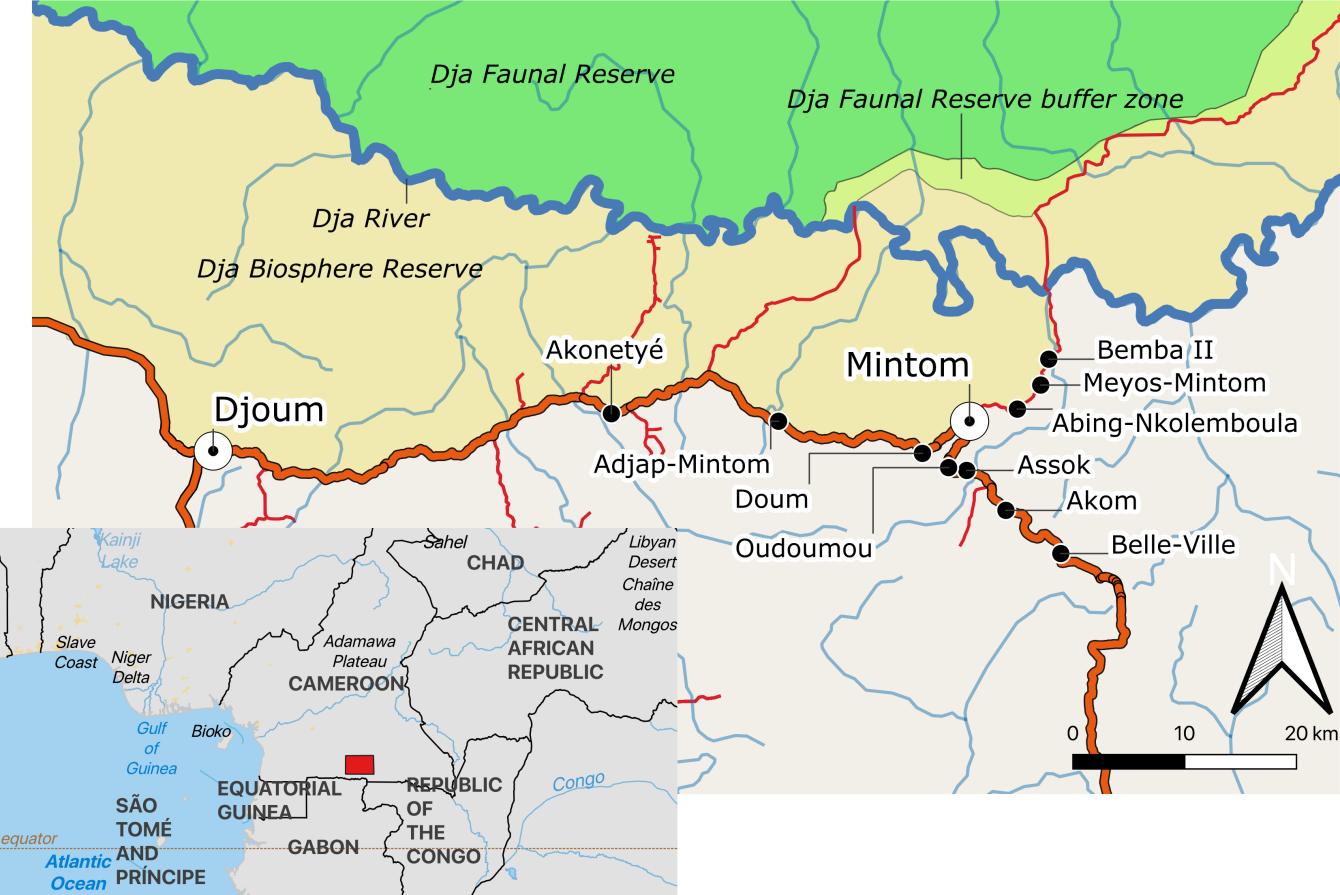
Map of the study area indicating the location of the ten study villages in southeastern Cameroon. Map created from public domain map dataset from Open Street Map, diva-gis (diva-gis.org) and Natural Earth (http://www.naturalearthdata.com). The Southern border of the Dja Biosphere Reserve was mapped following Bruce et al. (2018).

This study was conducted in 10 Baka villages, located along the Djoum-Mintom road south of the Dja Faunal Reserve (DFR) and bordering the Dja Biosphere Reserve in southeast Cameroon (Fig. 1). Bemba II is the village nearest to the DFR and Belle-Ville is the village furthest away (shortest bee-line distances of around 17 km and 30 km, respectively). From population censuses conducted by us in all ten villages (Table S1), we counted a total of 237 dwellings (largely poto-poto houses but also mungulu huts), of which 172 (72.57%) were lived in during the study. There was a mean (±SD) of 4.33 ± 2.77 (range 1–17) persons (men, women and children of differing ages) living in the occupied dwellings. Vacant dwellings belonged to families that were in forest at the time of the study. Total village population sizes ranged from 25 persons in Meyos-Mintom to 111 in Akom. Villages inhabited by Bantu-speaking farmers were also found on the Djoum-Mintom road. The relationship between farmers and Pygmies in the study region is similar to that reported for other rainforest areas of central Africa (see Rupp, 2003; Van de Sandt, 1999; Matsuura, 2011; Oishi, 2012). These studies have shown that farmers and Pygmy hunter-gatherers share a mutually dependent relationship with regard to lifestyle and culture but have ambivalent attitudes about one another characterized by both discrimination and respect (Hattori, 2014).

Agriculture alongside the harvest and trade of non-timber forest products are practised by Baka and Bantu-speaking farmers but to different degrees. Baka have gradually become sedentarised since the 1950s (Althabe, 1965; Kitanishi, 2003; Leclerc, 2012). After relocation from the forest, Baka started growing crops such as plantain, banana, and cassava. Subsistence farming has increased in recent years in the studied villages, particularly as a result of agricultural programmes initiated by our partner Zerca y Lejos (ZyL) (Zercay Le jos, 2020a; Zercay Le jos, 2020b), a Spanish NGO working on development and health support to Baka communities in the region. The result of this has been that fewer Baka work directly for the Bantu-speaking farmers on “petit jobs” such as clearing forest for planting, planting and harvesting crops. Although we did not observe any major conflicts as a result of these changes, in western Cameroon Van de Sandt (1999) reported that community divisions between Bagyeli and Bantu-speaking farmers were exacerbated when the former group adopted farming, leading to the seizure of Bagyeli land by farmers. Baka, however, are socio-economically more advantaged, compared to other Pygmy groups such as the Bagyeli (Kitanishi, 2003).

The terrain of the region is sloping with gently rolling hills. Altitude varies from 250 to 800 m and averages 600 or 650 m. The climate of the region is characterized by a four-season equatorial climate. A major dry season is from December to March, a minor rainy season from March to June, a minor dry season in August, and the major rainy season from September to December. Rainfall recorded for Djoum averages 1,500–2,000 mm per year, and some precipitation is common even during the dry seasons (World Weather Online, 2020). Average temperature remains fairly steady year-round, averaging 25 °C, fluctuating slightly with the seasons. The long, rainy season is the coldest time of year, and the long dry season the warmest. Humidity is high and does not vary throughout the year.

The major vegetation type is a mixture of evergreen and semi-deciduous forests (Letouzey, 1985). The forest is degraded alongside the dirt roads and main road, due to pressure from housing and agriculture. Logging operations in the region started in the 1970s as far of the extension of operations into the last remote tracts of intact forest in the east and south of the country (Global Forest Watch, 2000). Logging roads has enabled the connection of villages to market areas and to the main towns of Mintom and Djoum (Fig. 1).

Hunts take place at a distance from the village (Fa et al., unpublished data, 2017–2018). Women also hunt but this is much more localized, around their farms. Baka farms are relatively small patches of land dedicated largely to the cultivation of subsistence crops such as cassava, plantain and bananas. Hunting territories extended up to the Dja River, but never overlapped with the DFR (Fa et al., unpublished data, 2017–2018).

### Data collection

In following the principle of free, prior and informed consent (FPIC), allowing the studied communities to give or withhold consent to our project, we (E.A.M., G.R.B., R.O.) first organized several meetings with each village. During these meetings we asked the participants to highlight the main challenges faced in the daily lives; they identified key problems around agriculture and hunting. To understand the issues affecting hunting, we recruited hunters in each village to volunteer to self-report their daily hunting activities over a period of 5 months.

All hunters freely participated in our project without remuneration, gave verbal consent to do so, and could stop contributing to the project if they so wished. Hunters were informed that their identity would be kept anonymous and all information provided would be treated confidentially. No informant under the age of 18 (minors according to Cameroon law) were involved in our study. Importantly, we emphasised that our project was not focusing on the illegality of hunting (i.e., poaching) but to understand hunting as a subsistence activity. In each village, we recruited a reporter to oversee data collection from participating hunters. These village reporters (VR) were given a mobile telephone to allow them to communicate with the field team when needed and were paid 25,000 CFA (around 40USD) per month.

VR were all Baka schoolteachers (hence literate) who worked for ZyL’s education programme (Zercay Le jos, 2020b); they were full-time residents in each village. All VR were trained by R.O. in data collection methods in Fang; the Fang language being the *lingua franca* used between Baka and Bantu-speaking farmers in our study area. The VR visited every participating hunter’s household at the end of each day and hunters were asked to provide information for each returned carcass: 1) species, 2) hunting method used, and 4) whether the quarry was to be eaten at home, the complete carcass sold, or parts of the animal consumed and other parts sold. The unit of reporting was each hunting trip completed at the day of recall (rather than a 24 h recall), as some trips lasted for two or more days. The VR also noted the start and end of each hunting day by asking the hunter the time he/she left and returned to the house (if they possessed watches) or the approximate time taken as the mid-point in one of six pre-determined periods of the day (Grand matin—05.00–06.00, Matin—07.00–11.00, Midi—11.30–12.00, Après midi –12.00–15.00, Soir—15.00–20.00, Nuit—20.00 onwards). No-hunt days were also recorded. All VR communicated with hunters in Baka and information was recorded onto pre-prepared data sheets.

Species names were given in Baka by the hunters and scientific names were assigned according Kingdon et al. (2013) for mammals, Borrow & Demey (2008) for birds and Chirio & LeBreton (2007) for reptiles. Species hunted were classified according to their threatened status in the IUCN (2020), and according to the three protection classes (A, B, C) within the Cameroon Forestry & Wildlife Law (Republic of Cameroon, 1994).

Checks to ensure the correct application of methods, and to collect completed forms, were undertaken weekly by one of our field team members (R.O.), with supervisory visits by R.O.B. and E.A.M. All three team members were permanently based in the study area and were headquartered in Djoum.

Hunting practices were recorded every day by the VR in the 10 study villages over a complete period of five months each. Sampling was organised into two phases: a first Phase during Mar. to Jul. 2018 in which Bemba II, Abing-Nkolemboula, Doum, Assok and Belle-Ville were covered, and a Phase 2 (Oct. 2018 and Feb. 2019) in which the other five villages (Akonetyé, Adjab-Mintom, Meyos-Mintom, Odoumou, Akom) were visited. We selected villages to sample in each Phase so as to achieve a broad geographical spread of settlements i.e., not clustered, in each of the two phases.

Ethical approval was not required in this study, although it meets the guidelines of the Social Research Association (2003). Permission to undertake field work in our study area was granted by the Ministry of Scientific Research and Innovation (MINRESI), via the Center for International Forestry Research (CIFOR) in Cameroon. Authorisation to work with human subjects was covered by the Arrete No. 00034/A/MINATD/DAP/SDLP granted by the Ministere de L’Administration Territoriale et de La Decentralisation of the Government of Cameroon to ZyL.

### Statistical analyses

We tested skewedness of the data using Spearman’s rank correlation tests and the Jarque–Bera normtest (Jarque & Bera, 1987; Gavrilov & Pusev, 2015).

To evaluate the variables impacting the proportion of hunting trips that failed to produce any carcasses we used a general linear model, GLM, with a binomial error distribution. Variables were: the field effort deployed by each hunter in terms of hunting effort in hours, the hunter’s overall experience as estimated by the total hunting effort and hunting return of carcasses over all hunting trips, and the village characteristics as estimated by the total hunting return of carcasses weights in kg by all village hunters, the average return of carcass weights by each village hunter, the number of carcasses returned by all village hunters and the percentages of hunting with rifles, traps and dogs. Carcass weights were estimated from published mean weights of mammals (Kingdon et al., 2013) and birds (Dunning, 2007), and reptiles (Chirio & LeBreton, 2007).

To evaluate the diversity of hunted animals by village, we applied the following diversity metrics (Magurran, 1988; for further details see Table S2): (1) Species richness; (2) Dominance; (3) Simpson’s index; (4) Shannon–Wiener index; (5) Evenness and (6) Chao 1. For all diversity metrics, we calculated the 95% upper and lower confidence intervals by 10,000 bootstraps, each with the same number of individuals as in each original sample. For generating bootstraps, the random samples were taken from the total of all columns, and the taxon was chosen with probabilities according to the original pooled abundances.

To compare animal biomass extracted in each study village with other hunting studies in the Congo Basin in Fa et al. (2016), we calculated: (1) *extraction rate*, as the mean number of kilograms of wild meat extracted by a hunter (H) in a year by multiplying the average amount of wild meat (kg) taken per hunter (kg H^−1^ yr^−1^) by the number of occupied households (since there was a hunter in each household) in the village; (2) *harvest rate*, as the total number of kilograms of wild meat extracted per inhabitant in each village per annum (kg P^−1^ yr^−1^) calculated from 1) divided by the total number of inhabitants in each village. This index measures per capita yield of game animals for the average person (P) in a community in one year. To calculate the harvest rate, we assumed, based on our field observations, that there is one hunter per household. Data on numbers of households and numbers of inhabitants per village stem from anthropological surveys (see above).

To better visualize the amount of meat extracted per village, we translated the weight of wild meat estimated for each into cattle meat equivalents, as used by Nasi, Taber & Van Vliet (2011) to highlight the level of substitution needed if wild meat was to be replaced by domestic meats. We choose adult UK dairy cattle, which weigh on average 617 kg, only slightly more than Argentinian, Canadian, Danish, Dutch, German and Swiss dairy cattle (600 kg), but less than French (650 kg), US (680 kg) but more than New Zealand (458 kg) and the assumed ‘standard average’ for all breeds across 10 European countries (500 kg) (Schubert et al., 2019).

Statistical analyses were conducted using the R statistical environment (R Foundation for Statistical Computing, 2016) and Past 3.0 (for diversity analyses; Hammer, 2019), with all tests being two-tailed. Alpha level was set at *P* = 0.05.

## Results

### Hunting effort and hunting returns

Excluding 68 hunting trips with no information on hunting returns, we obtained data on 1,946 hunting trips by 121 hunters (118 men, 3 women) from the ten study villages (raw data in Table S3). Assuming that there was at least one male hunter in the 172 occupied houses (see Table S1), we then sampled 77% (*N* = 133) of hunters in the studied villages.

**Figure 2:**
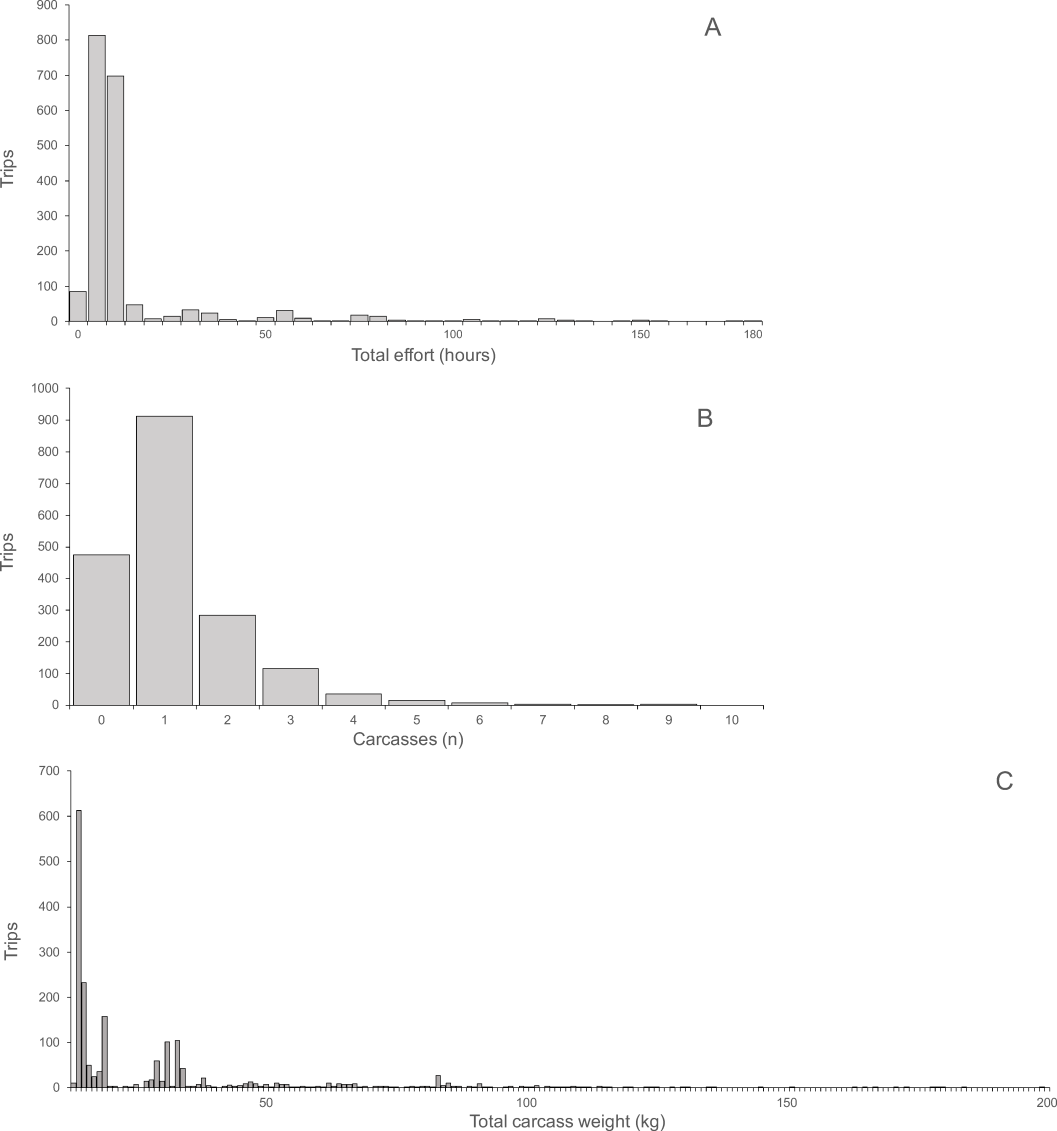
(A) Total hunting effort, (B) Number of carcasses, (C) Total carcass weight.

Hunting effort, expressed as the duration of hunting trips, varied between one and 180 h and was skewed with a mean of 13.17 h and a median of eight hours (Fig. 2A). For each hunting trip, a mean ± SD of 1.15 ± 1.11 carcasses (median 1, range 0–9) were returned (Fig. 2B). Total carcass weight returned from trips was skewed with a mean of 15.0 kg, median of 6.3 kg and range of 0.3 to 148.3 kg (Fig. 2C). Hunting effort and number of returned carcasses were positively correlated indicating both, a significant linear relationship and a large variance with only 10.8% of the variance explained (Spearman’s *r* = 0.33, *P* < 0.0001, *N* = 1, 946). The variance occurred between trips of each hunter and between hunters, as indicated by the larger proportion of variance explained (67.3%) by the correlation between total hunting effort and returned carcasses for all hunters (Spearman’s *r* = 0.82, *P* < 0.0001, *N* = 121). The average hunting effort versus the average number of returned carcasses across all hunters in the ten villages was positively correlated, explaining 73% of the total variance (Spearman’s *r* = 0.85, *P* < 0.004, *N* = 10). The average number of returned carcasses and the average time spent to hunt each carcass was negatively correlated across villages explaining 48.6% of the total variance (Spearman’s *r* = 0.70, *P* < 0.031, *N* = 10). In other words, the higher the number of animals successfully hunted the smaller the proportional time required hunting each animal.

About a quarter of trips (23.4%) failed to return a carcass. As expected, failure was negatively correlated with hunting effort (GLM coefficient = − 0.015, Standard error = ±0.005, *P* < 0.0001). Failure was also significantly negatively correlated with the hunter’s total return of carcasses (−0.030 ± 0.005, *P* < 0.0001) and significantly positively correlated with the total hunting effort (0.003 ± 0.0005, *P* < 0.0001). At a village level, failure was significantly negatively correlated with average village return weight (−0.191 ± 0.025, *P* < 0.0001), average village return rate (−0.871 ± 0.305, *P* = 0.004) and the proportion of dogs used in hunts (−0.135 ± 0.020, *P* < 0.0001), but significantly positively correlated with average hunter’s return weight (0.009 ± 0.001, *P* < 0.0001) and percentage gun hunting (0.022 ± 0.010, *P* = 0.030). The parameter of percentage snare hunting was not significant (0.003 ± 0.010, *P* = 0.74).

**Figure 3:**
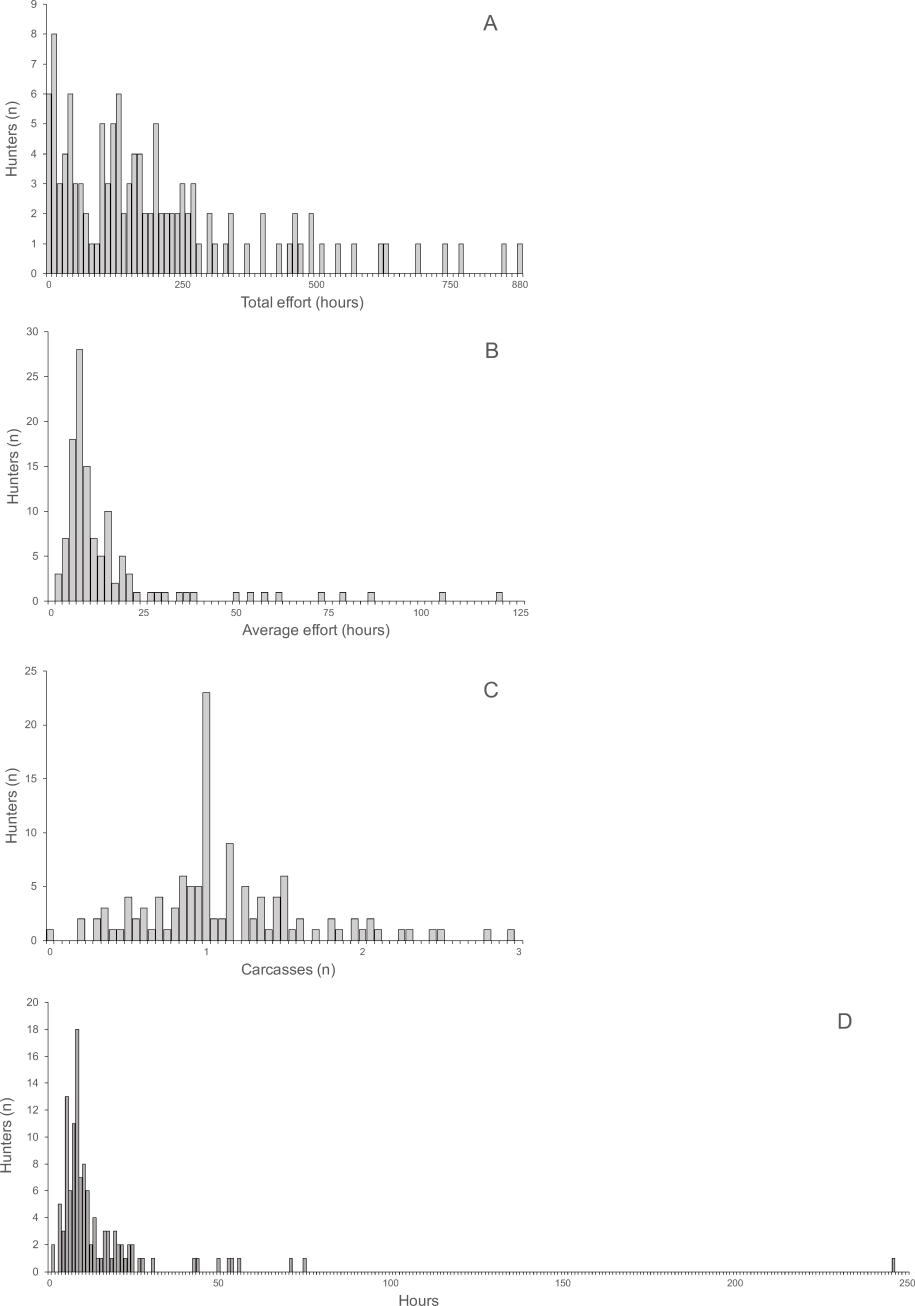
(A) Total hunting effort, (B) Average hunting effort, (C) Average number of returned carcasses, (D) Average effort required to successfully hunt one animal.

The distributions of total and average hunting effort, the average number of returned carcasses and the average effort required to successfully hunt one animal were all highly skewed (Fig. 3). The skew of the visually least skewed distribution of the average of returned carcasses per hunter was significant (Jarque–Bera normtest, *T* = 0.91, *P* < 0.0001).

Average return over hunting trips and hunters per village ranged from 0.61 to 2.06 (mean ± SD 1.15 ± 0.43) and was not skewed (Jarque–Bera normtest, *T* = 0.89, *P* = 0.12). Hunting effort per hunter was skewed (*T* = 1.49, *P* = 0.014) and ranged from 125.6 to 357.1 h (mean 213.7 h, median 180.12 h). The average time required to hunt one carcass was also skewed (*T* = 1.49, *P* = 0.014) and ranged from 6.6 to 26.9 h (mean 12.5 h, median 10.7 h). The average return was significantly correlated with the average time spent on hunting trips by hunters (Spearman’s *r* = 0.85, *R*^2^ = 73%, *P* < 0.004, *N* = 10). Average return per hunter and average time per carcass were significantly negatively correlated (Spearman’s *r* =  − 0.70, *R*^2^ = 48.6%, *P* < 0.031, *N* = 10).

### Species composition

As many as 49 separate species (Mammals = 37; Birds = 6; Reptiles = 6) of 24 families (Mammals = 16, Reptiles = 5, Birds = 3) were hunted in the study; 2,245 carcasses in total, out of which 68 carcasses (3%) were not identified to species (Full species list, Table S3). No songbirds were hunted, but large birds, hornbills and guinea fowls, were taken. Seven species of the mongoose family, one cat, one mustelid and one palm civet were noted in the study. Amongst the primates, eight monkeys, one ape and a prosimian were hunted. As many as nine bovids, one member of the pig family and one Tragulid represented the hunted ungulates. Only three rodents and one unidentified squirrel were recorded.

**Figure 4:**
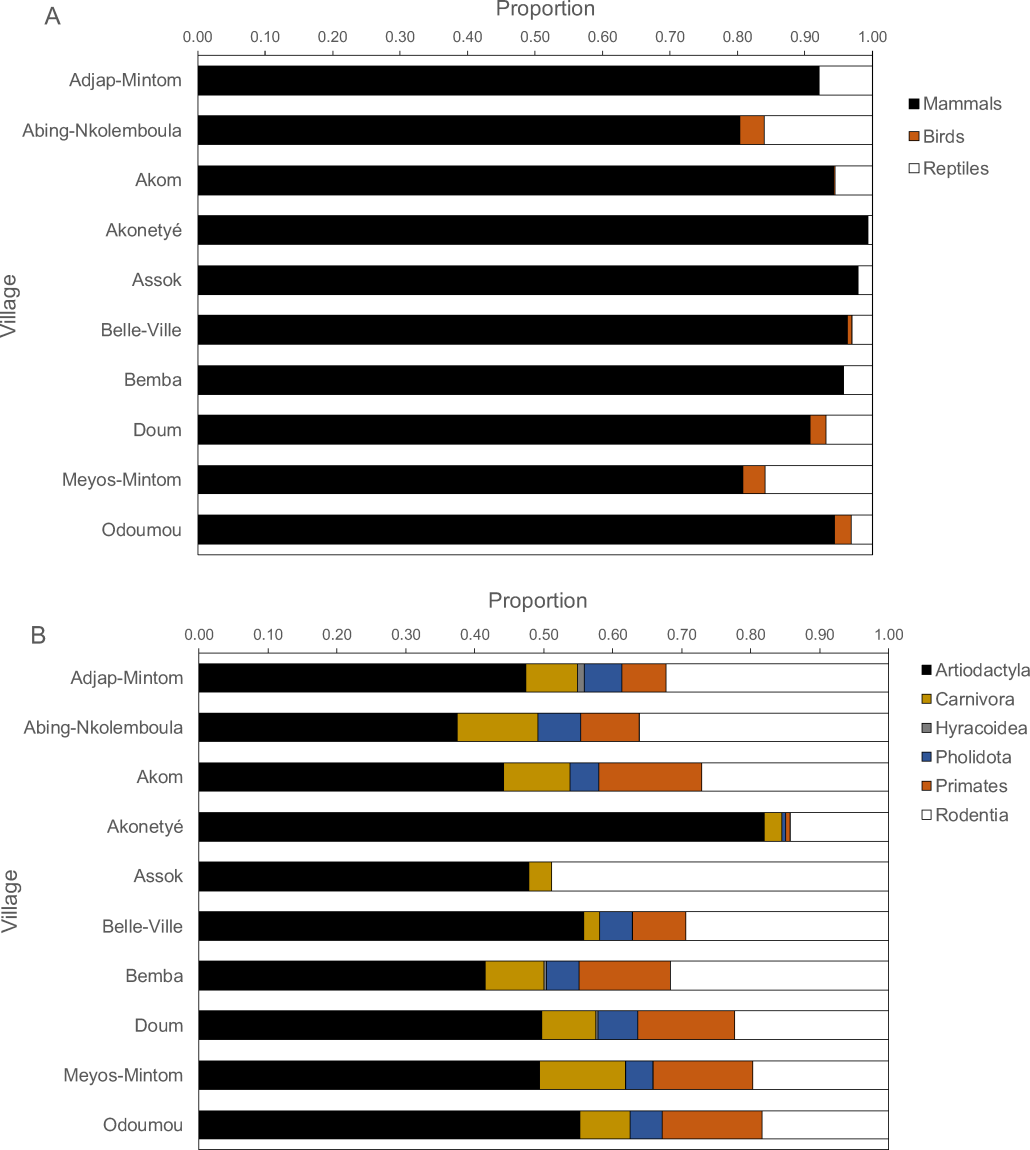
Proportions of carcasses by (A) animal classes and (B) mammalian orders hunted across 121 hunters (*n* = 1, 946 hunting trips) in the ten Baka study villages in southeastern Cameroon. The dotted lines solely aid visually grouping animals and are not.

By village, mammals contributed between 80% and 100% of all carcasses, but more reptiles were hunted than birds (Fig. 4A). In only two villages, reptiles and birds constituted about 20% of returns, in all others less than 10%. Amongst mammals, ungulates contributed the highest proportion of mammalian returns (*χ*^2^ test on the data frequencies, *P* < 0.001), followed by rodents with two exceptions where ungulates and rodents were equal (Fig. 4B). In all villages, more ungulates were hunted than rodents (ungulate: rodent ratio, 2.2 ± 1.4), ranging from 0.98 in Assok to 5.74 in Akonetyé. Hunted ungulates ranged between 30% and 55% with one notable exception of about 80% in one village (Akonetyé). Spearman’s rank correlations of mammal orders, birds and reptiles versus hunting methods (rifle, traps, dogs and others) were significant in only two cases, namely for primates for rifle hunting (Spearman’s *r* = 0.65, *P* = 0.049) and snare hunting (Spearman’s *r* =  − 0.73, *P* = 0.02).

### Species richness and diversity indices

**Figure 5:**
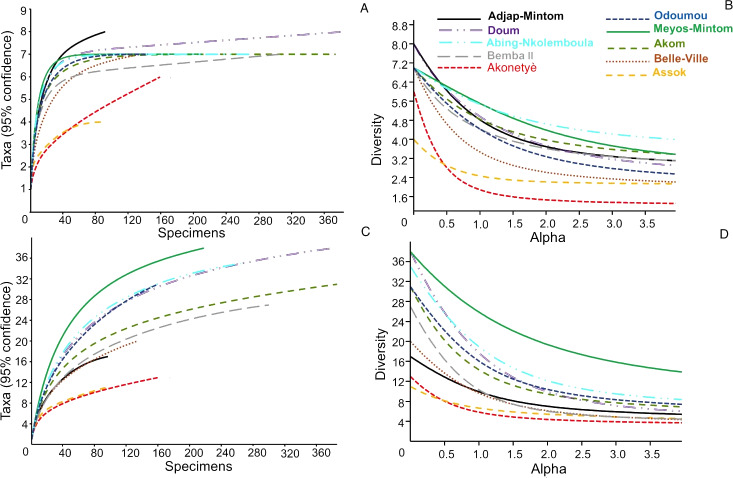
Diversity indices across the 10 villages. (A, B) Diversity profiles (95% confidence, after 9,999 bootstraps); for the community diversity of hunted animals in surveyed villages. (C, D) Saturation curves (with 95% confidence intervals after 9,999 bootstraps) for the community diversity of hunted animals in surveyed villages.

The summary of the diversity metrics calculated for each community of hunted animals by village is presented in Table S5. Scrutiny of the diversity, evenness and dominance indices (and of their confidence intervals) at species groups and species clearly revealed that the hunted animals’ community characteristics were similar among villages, with however one exception: the village of Meyos-Mintom. In this village, the species diversity and evenness were higher, and the dominance index was lesser, than in all other villages. This result is fully confirmed by a diversity profile analysis (Figs. 5A, 5B), with the curve for Meyos-Mintom standing alone and significantly higher (*P* < 0.01 at ANCOVA) compared to the curves of all other villages. Chao-1 estimates (Table S5) showed that the number of hunted species recorded during our study was significantly lower than that expected for most of the surveyed villages (i.e., Akom, Akonetyé, Assok, Belle-Ville, Doum, Meyos-Mintom and Oudoumou), and a saturation curve analysis (Fig. 5B) established that in all villages a plateau phase in the number of hunted species was not reached. Even by this analysis, Meyos-Mintom appeared the village where the plateau phase was the furthest from being reached, and therefore where our sampling appeared the least accurate (given that the diversity of species is highest there).

### Biomass extracted

A total of 53 carcass types, i.e., distinct identified or unidentified species, were reported by hunters. Of these, 44 could be determined to the species level, two only to the genus level (one duiker and one pangolin) and two to family level (Herpestidae and Phasianidae, respectively) (Table S3). Five reported carcass types (a “bird”, “monkey”, “squirrel”, “snake” and “animal”, respectively) could not be identified because the carcasses were sold prior to the VR’s visit. The mean weight of the 46 species could be estimated according to the literature, representing 95% of all carcass types, whereby the weights of the two possible Phasianidae species and the two possible pangolin species were averaged since the species could not distinguished separately (Table S3).

The accumulated total weight of the 46 species with weight estimates was 20,609 kg across the ten villages. The average return per hunter varied between 52.3 kg and 389.1 kg among villages (mean ± SD = 183.7 ± 115.6 kg, skew not significant with *T* = 0.369, *P* = 0.48). By species, just over half of the animal biomass extracted by all hunters in the studied villages was provided by four mammal species: the blue duiker *Philantomba monticola* (17.99%), brush-tailed porcupine *Atherurus africanus* (15.10%), bay duiker *Cephalophus dorsalis* (10.07%), and Emin’s pouched rat *Cricetomys emini* (7.93%).

Estimates of mean ± SD harvest and extraction rates per annum (from the average amounts per village per sample month multiplied by 1) for all villages pooled were 124.0 ± 118.1 kg P^−1^ yr^−1^ (median = 69.4) and 440.8  ± 277.4 kg H^−1^ yr^−1^ (median = 380.5), respectively. By village, a mean ± SD of 7,569.7 ± 6,103.4 kg yr^−1^ of wild meat were extracted per annum, ranging from around 2,080.8 kg in Belle-Ville to 19,351.4 kg in Akom (Table 1). Mean body mass of animals hunted in all villages was 10.1 ± 4.0 kg, lowest (6.5 kg) in Abing-Nkolemboula and highest (16.9 kg) in Akonetyé (Table 1).

**Table 1:** Summary of volume of prey biomass taken by hunters and mean body mass recorded in each of the 10 study Baka villages in our study in southeastern Cameroon.

Village	Mean prey biomass recorded per hunter per village	Number of hunters contributing data per village	Extraction rate (kg H^−1^ yr^−1^) per village	Total prey biomass extracted per village	Harvest rate (kg P^−1^ yr^−1^) per village	Mean body mass (kg) of hunted species
Adjap-Mintom	88.5	8	212.5	3186.8	59.0	7.2
Akom	336.0	9	806.3	19351.4	208.1	7.8
Akonetyé	210.8	13	506.0	11637.6	104.8	16.9
Assok	52.3	12	125.5	2887.4	27.2	7.0
Belle-Ville	86.7	13	208.1	2080.8	27.4	9.2
Bemba II	88.5	22	212.5	2974.4	72.5	6.7
Doum	267.8	11	642.7	15425.9	248.8	9.5
Meyos-Mintom	389.1	9	933.8	4669.0	42.8	15.2
Abing-Nkolemboula	135.8	12	326.0	3911.7	66.3	6.5
Odoumou	181.3	13	435.1	9572.2	382.9	14.7
Mean ± SD	183.7 ± 115.6	12.2 ±3.9	440.8 ± 277.4	7569.7 ± 6103.4	124.0 ± 118.1	10.1 ± 4.0

### Hunting methods

Most animals were trapped (65.77% ± 16.63) using snares; these consist of a noose anchored to the ground, made usually of wire or a strong string. A lower proportion of animals were killed with single-barrelled shotguns (22.56% ± 17.72) but other methods (8.69% ± 6.96) such as hand collection (for tortoises in particular) and dogs (2.96% ± 4.49) were employed. By village, the highest percentage of animals trapped was in Akonetyé (99.4%), but highest percentage of animals shot was in Odoumou (50.9%). The highest percentage of animals hunted with dogs was in Bemba (37%), followed by Akom (34%); in the other villages only between 0–6%.

**Table 2:** Average number of animals killed per hour hunting according to the different hunting methods used and village. The total number of hunting trips was 1946.

	**Gun**	**Trap**	**Other methods**	**Dogs**	
**Village**	Mean ±SD	Mean ±SD	Mean ±SD	Mean ±SD	**N**
Adjab-Mintom	0.04 ± 0.07	3.61 ± 2.49	1.13 ± 0.78	0.00	148
Akom	0.24 ± 0.24	51.20 ± 41.63	5.94 ± 4.83	0.79 ± 0.64	243
Akonetyé	0.00	0.21 ± 0.10	0.00	0.00	174
Assok	0.00	0.00	1.05 ± 0.88	0.09 ± 0.08	78
Belle-Ville	0.04 ± 0.06	7.52 ± 4.77	0.84 ± 0.53	0.00	221
Bemba	0.06 ± 0.03	11.60 ± 5.95	2.32 ± 1.19	0.43 ± 0.22	295
Doum	0.36 ± 0.40	40.06 ± 50.97	4.13 ± 5.25	0.00	185
Meyos-Mintom	0.07 ± 0.07	10.23 ± 6.84	6.61 ± 4.42	0.13 ± 0.08	189
Nkolemboula	0.02 ± 0.02	4.03 ± 2.61	17.36 ± 11.24	0.09 ± 0.06	262
Odoumou	0.09 ± 0.13	6.52 ± 6.79	1.45 ± 1.51	0.02 ± 0.03	151

**Figure 6:**
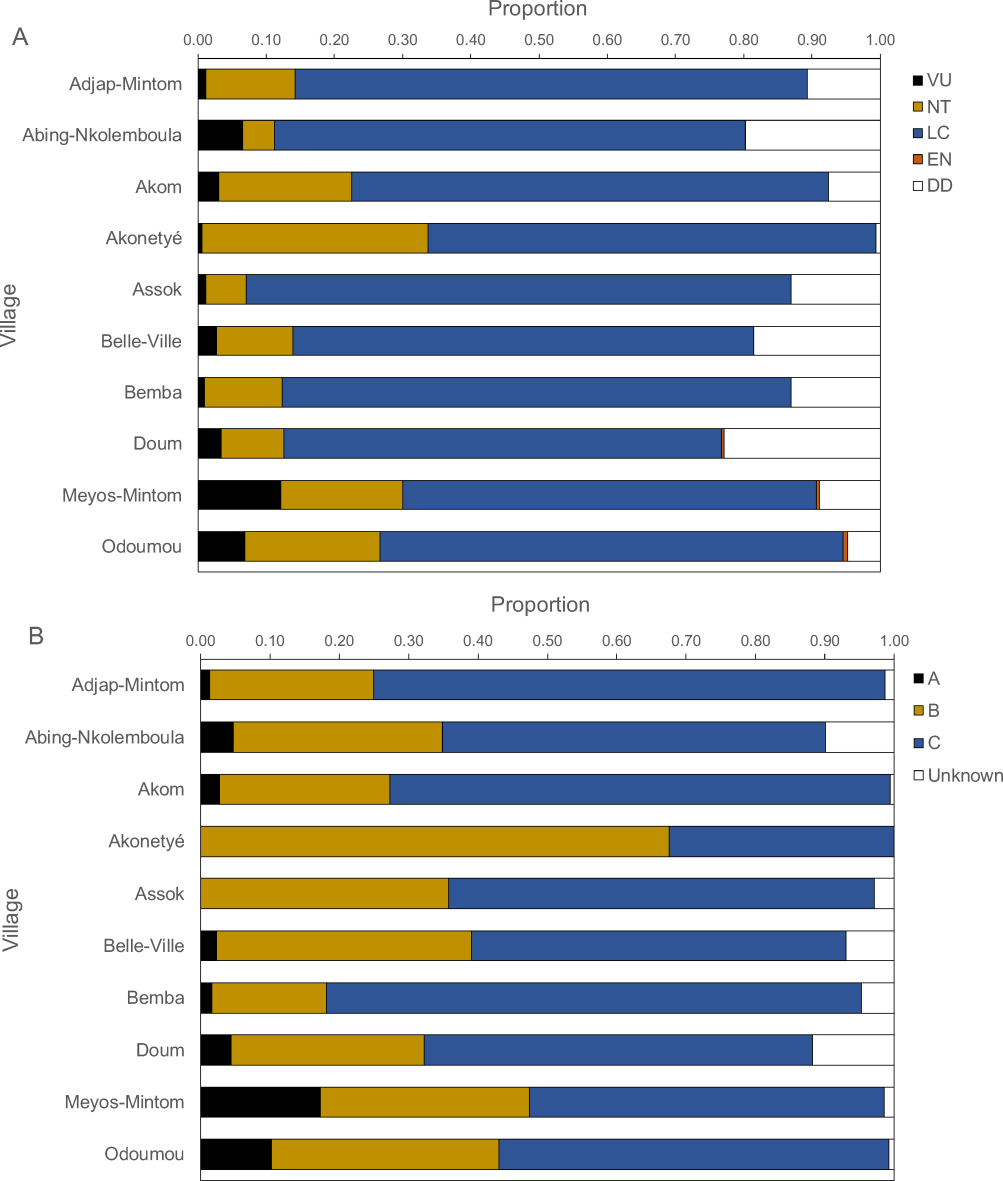
Proportions of IUCN red list categories (A) and Cameroonian legal status (B) of carcasses.

Calculated efficiency of the different hunting methods in terms of animals killed per hour hunting, taken from recorded hunting trip duration, shows that the average returns for trapping was considerably more efficient (15.15 ± 27.80 animals/hr) than other methods (4.80 ± 7.34 animals/hr), dogs (0.19 ± 0.36 animals/hr) or hunting with guns (0.10 ± 0.19 animals/hr). Differences in hunting returns by method varied between villages, with gun hunting being more efficient in Doum, trapping returns and hunting with dogs highest in Akom, and other methods in Nkolemboula (Table 2). Trapping returns correlated with gun hunting (Pearson’s *r* = 0.91), dogs (*r* = 0.64) and other methods (*r* = 0.13).

### Destination of carcasses

Most animals hunted in all villages were consumed by the hunter and his family (48.07% ± 17.58) The mean number of carcasses sold in all villages was 32.73% ± 12.55, followed by a lower percentage of carcasses partially sold and consumed (19.21% ±17.02). The highest proportion of animals sold in a village was in Assok (50.5%) and lowest in Belle-Ville (14.3%). Most carcasses were sold to neighbours (41.9%) and resellers (31.3%), the rest to passersby. Animals consumed by the hunter were lowest in Akonetyé (6.9%) and highest in Bemba (69.0%).

### Threatened and legal status of hunted species

Figure 6 presents the IUCN Red List categories and the Cameroonian legal status of hunted species. Between 60% and 80% of carcasses belonged to the “least concern” category, followed by “near threatened”, “vulnerable” and, rarely “endangered”. The “near threatened” category was represented between 5% and 20% with the exception of Akonetyé, where over 30% of carcasses belonged to this threat category. “Endangered” species were represented only in Odoumou where 1% of carcasses belonged to this group. The only endangered species was the central chimpanzee (*Pan troglodytes*). The most frequent Cameroonian legal status was “Class C”, followed by “Class B” with the exception of Akonetyé where more carcasses belonged to “Class B” than “Class C”. Meyos-Mintom and Odoumou had the highest proportions of “Class A” carcasses with 17% and 10%, respectively. All other villages had a frequency of 0% to 5% of “Class A” animals.

## Discussion

By involving the participation of an estimated 77% of potential hunters in the ten study villages, we were able to amass a significantly large volume of information over a short time period. We recorded not just information on numbers of animals hunted but also on the time invested in hunting trips (over 1,000 recorded). Each village was monitored daily over a five-month period according to the budget available for this activity within the main project. The latter had various work pillars, one of which was the assessment of extraction of wild meat by the Baka in sample villages.

Given that we opted for a broader, short duration and very intensive approach for the study we necessarily had to sacrifice some precision in some aspects of our work because of the cost and extra time that would have incurred by providing hunters with balances and watches to exactly measure biomass and time spent for hunting. We are aware that the estimation of biomass extracted could have been affected by the fact that we used average body mass (adult male and adult female) of hunted species from the literature, which does not take into account the proportion of juvenile animals. The percentage of juveniles in our study may have varied by species, hunting history of the site, as well as hunting method. For primates, Kümpel (2006) showed that more juveniles of larger (mandrill) than smaller species (guenons) were shot because juvenile mandrills are still a sizeable prey. Noss (2000) observed a greater proportion of female and juvenile duikers snared than found in wild populations in the Central African Republic perhaps reflecting the state of depletion of these populations, as shown for blue duikers in Bioko (Grande-Vega et al., 2016). In the case of loss of precision in recording length of hunting trips, there may have been some underestimation of <5 h. The impact of this is likely to be relatively small. Because each village was only followed for five months some seasonality may have been missed. However, since villages were monitored primarily during the rainy seasons when most of the hunting takes place, we may have captured most of this activity; dry seasons are mostly dedicated to fishing as shown by Hattori (2014) and Duda (2017) for other Baka villages. We acknowledge that some of these limitations can be overcome in longer studies in which e.g., body mass of actual hunted prey can be gathered or more time and resources invested in training local participants in the use of recording equipment such as handheld computers attached to global positioning systems (GPS) as in Lewis (2012). The limitations in weighing each carcass exactly and in measuring the exact length of hunting trips are an epiphenomenon of the complexity of precise data collection on some activities when conducting field work in many settings.

Our work adds to the literature on Baka hunting in southeastern Cameroon but focuses much more on the ecological analyses of hunting in sedentarised villages. We show that it is possible to generate large amounts of useful data in a short space of time. Although longer term studies may complete the hunting offtake picture of these communities we suggest that shorter term, more intensive data collection with the support and help of hunters themselves, can be a cost-effective way of assessing the exploitation of wild meat resources. All hunters involved in our study were fully aware of the purpose of our research, which ultimately aimed not just to quantify wildlife extraction by their communities, but ultimately determine ways in which they can regulate their hunting activities in pursuit of more sustainable goals. Duda (2017) stresses that the establishment of trust with informants is essential to reduce reporting bias. Considering that some of us lived in the region, spoke the local language, that local village reporters were a conduit of information flow with informants and that one of the organisations involved in this study, ZyL, has been working with the studied villages providing health service for over a decade (Zercay Le jos, 2018; Funk et al., 2020), we are confident that underreporting of species prohibited by law was likely to be low if it occurred at all. In our briefings of the participating hunters we emphasised that hunting should be carried out as normal and reassured them that there would be no reporting to the authorities.

Similar to other Pygmy hunters throughout the Congo Basin (Fa et al., 2016) mammals contributed almost all hunted carcasses in the studied villages, followed by reptiles and least birds. Amongst mammals, ungulates comprised the highest proportion of returns. Snaring was commonly used to catch most prey species, but shotguns were only important for hunting primates. Animal kills per hour hunting for the different methods showed that trapping was by far the most productive, followed by other methods and dogs. Lowest kill returns were recorded for gun hunting. Explanations of these differences as well as the contrasts between villages may be a combination of prey availability and hunter proficiency. However, the positive correlations between the returns of all methods indicate that hunters are not using any particular method preferentially.

As in other hunting studies of Baka communities in southern Cameroon (e.g., Duda, 2017; Duda, Gallois & Reyes-Garcia, 2017; Duda, Gallois & Reyes-García, 2018; Duda & Gallois, 2019), our study shows that most hunters engage in short hunting events, target a low diversity of relatively abundant species such as small duikers, and large rodents (porcupines and rats), and hunt less larger-bodied species. These game profiles are also similar to other sites throughout the Congo Basin (Bennett & Robinson, 2000; Fa & Brown, 2009; Fa et al., 2016).

We described species richness and diversity of hunted animals in each village using a variety of indices commonly used in the ecological literature. We showed that the community characteristics of hunted animals were similar among villages, with the exception of Meyos-Mintom which stands alone in terms of exhibiting a higher species diversity and evenness, though with a lower dominance index, than the other villages. The similarity of the species hunted in each village can be explained by the fact that most hunters are using similar habitats, close to their villages. These habitats are likely to have the same faunal assemblages and abundance. Moreover, the lack of significant differences in what species are hunted and numbers of these taken attests to the fact that, overall, hunters in the studied villages are practicing similar hunting techniques. The distinctiveness of Meyos-Mintom may be explained by the fact that hunters from this village may be using hunting territories that are less depleted, and therefore currently more diverse. Further explanations for the difference are not possible at present since more field surveys of faunal profiles and abundance are required. Since the “prey composition” of the various villages is similar, some competition among hunters may arise and increased impacts on prey communities could occur. Further study of these issues is needed, and ways of resolving social clashes or any social insecurity due to “hunting competition” must be alerted.

Using data for carcasses which could be identified to species, and for which we could obtain body mass data, we estimated that around 50,000 kg of wild meat were extracted per annum by our participating hunters across the ten study villages. On accounting for the number of occupied houses in all the villages, we calculated that as much as 7,000 kg (range 2,000–19,000 kg) of wild meat were extracted annually per village. These figures represent as many as 12.3 ± 9.9 dairy cattle equivalents (weight of and dairy cattle 617 kg from Schubert et al., 2019) per village. The provision of such relatively large amounts of animal protein is fundamental for a people where meat from livestock rearing is absent. Moreover, hunting is a long-established part of their lives, of immense cultural value (Bahuchet, 1992; Köhler, 2005; Ichikawa, Hattori & Yasuoka, 2016; Duda, Gallois & Reyes-García, 2018) even as some authors suggest, “a religion” (Pemunta, 2019).

Comparisons of the game biomass calculated for the 685 villages with similar information from Pygmy and non-Pygmy sites in the Congo Basin indicate that hunting pressure from the studied villages is comparatively low. This indicates that Baka are probably not impacting the supply of wild meat in mid- to long-term if hunter densities do not increase. Whilst median amounts of game harvested per inhabitant per year in non-Pygmy and Pygmy groups was 233 kg and 212 kg of undressed meat from data in Fa et al. (2016), for the studied villages the median amount of game meat extracted per inhabitant was much less, 83 kg. Information on whether these amounts of wild meat (about 50 kg dressed meat, assuming that 60% of the carcass is usable, after skin and bones are removed) are sufficient to supply the daily needs of our Baka population is currently unknown, but our data compares favourably with the average annual amounts of meat consumed in the world of 43kg per person (Ritchie & Roser, 2017).

The opportunistic sale of wild meat by Baka families is a common practice, providing much of the monetary value of products sold (Duda, Gallois & Reyes-García, 2018). Our results also indicate that a large proportion of wild meat is consumed by the hunters and their families, with only a small amount being sold largely to neighbours and to some resellers. Resellers are traders from the nearby towns who travel to the Baka villages in search of meat to sell back in the towns.

Information on whether the extraction rates of the different species of game animals in the studied villages will require further data on actual population densities of the species. These data, as yet unavailable, will allow us to suggest whether the current hunting levels, which we know are below other studied areas, are above the production levels of the targeted. Given that most of the biomass extracted originated from highly hunting-resilient species namely the blue duiker, brush-tailed porcupine, bay duiker and Emin’s rat, current levels of hunting may be sustainable. Moreover, the mean body mass of prey taken in each village was comparatively high (average 10 kg with three villages above 15 kg), Also, because most hunted species were either of “Least Concern” in the IUCN Red List and belonged to Class C in the Cameroonian law, hunting in the studied villages are largely within the existing legislation. While prey species composition in other parts of southeastern Cameroon reflect differences in species abundance (Yasuoka, 2014), in most studied sites the most frequently captured animals are red duikers, and in particular Peter’s duikers (Class B) (Yasuoka, 2014). Animals of Classes A and B together accounted for 70–90% of the total catch in two studied villages in southeastern Cameroon (Ichikawa, Hattori & Yasuoka, 2016).

The main Cameroonian law regulating hunting (law No. 94/01 of January 1994 and Decree No. 95/466/PM of 20 July 1995) acknowledges user rights of local people to wild protein sources throughout the national territory, except on third party properties and protected areas (Djeukam, 2012). Traditional hunting is authorised for rodents, small reptiles, birds and other Class C animals exclusively for personal consumption and may not be sold. The latter restriction works against rural and indigenous groups fulfilling their family needs. This is because the sale of surplus meat can provide much needed income. However, although sale of wild meat is tolerated by many governments, the existing legal frameworks stigmatise hunters as criminals (Van Vliet et al., 2019). Thus, in the Cameroonian context, regulations on protected animals do not take into account the reality of actual hunting practices by local people. As Ichikawa, Hattori & Yasuoka (2016) argue, if they were to be strictly applied, it would seriously affect the livelihood of forest hunters who heavily depend on wildlife.

Our study has described hunting practices of sedentarised Baka groups in unprotected forest landscapes in the south of Cameroon. We do not quantify the energetic, macro- and micro-nutritional contribution that wild meat makes to the food security of our study communities. However, given that food security is determined not only by individual nutrients, but by availability, access, biological utilization, and stability of critical food resources (USAID, 1992), the observed extracted amounts of wild meat, which are mainly eaten at home rather than sold, can significantly improve household food security, as already demonstrated in other studies (see e.g., Friant et al., 2020). Unsustainable hunting and trade in bushmeat threatens stability of food security (Fa, Currie & Meeuwig, 2003), but wild meat extraction levels are likely to be sustainable in our study area if hunter densities do not increase as discussed above.

These communities are under huge political, economic, ecological and social pressures as they confront modern state laws and international development actors and agencies which could conflict with their ways of life (Pyhälä, 2012; Pemunta, 2013) but also inter-ethnic conflicts (Rupp, 2003). Issues resulting from the recent increase in the numbers of protected areas in the country may increase conflicts such as evictions, displacement and widespread multiple human rights violations as described by Pemunta (2014) and Ndameau (2001) and whose socioeconomic livelihood revolves mainly around hunting and gathering. The issue thus remains whether hunting be effectively managed to conserve biological populations while meeting human needs. If our study population is indicative of what may be happening in other forest areas in the south of Cameroon, we believe that the adequate management of game species must be sought by allowing rural communities to be able to continue to obtain natural resources in the same manner they are currently doing, but reinforcing these actions by a better understanding of traditional hunting regulations and sustainable management.

Despite that some Central African countries have recognized the rights of indigenous peoples in national law, Pygmies are still marginalised (Ohenjo et al., 2006; Wodon, Backiny-Yetna & Ben-Achour, 2012). With the development of new economic activities, Pygmy groups have witnessed the gradual reduction of access to forest resources, especially to game and edible wild plants. The expansion of protected areas has also contributed to the gradual reduction of access to forest resources (Ichikawa, 2006; Robillard, 2010; Lewis, 2012; Pyhälä, 2012; Yasuoka et al., 2015; Pyhälä, Orozco & Counsell, 2016). Although our study suggests that the Baka in the studied villages are able to extract sufficient amounts of wild meat from their current hunting territories it is likely that the presence of the DFR near the villages studied could have an effect on the mobility and hunting territories in the future. Further studies on year-round hunting activities using the techniques applied in this study, alongside spatial use of the landscape, are crucial to ensure that this vulnerable group of peoples are able to continue the lifestyle of their choice into the future.

##  Supplemental Information

10.7717/peerj.9906/supp-1 Supplemental Information S1 Supplementary Tables

10.7717/peerj.9906/supp-2 Supplemental Information S2 Raw data

10.7717/peerj.9906/supp-3 Supplemental Information S3 List of hunted species, IUCN Red List status and Cameroon law Class, and numbers of carcasses recorded for each village in the ten study villages in southeastern Cameroon

## Additional Information and Declarations

### Field Study Permissions

The following information was supplied relating to field study approvals (i.e., approving body and any reference numbers): Permission for field work was granted by the Ministry of Scientific Research and Innovation (MINRESI), Cameroon via the Center for International Forestry Research (CIFOR) in Cameroon.

### Data Availability

The following information was supplied regarding data availability: Raw data are available in a Supplementary Table.
