# Supplementary material for: Wild meat hunting and use by sedentarised Baka Pygmies in southeastern Cameroon
Source: PeerJ. 2020 Sep 17;8:e9906. doi: 10.7717/peerj.9906 (PMC7502248; doi:10.7717/peerj.9906)
Supplement: Supplemental Information S1 [file peerj-08-9906-s001.docx]

Supplementary Material

Supplementary Table 1. Population censuses carried in the ten study Baka villages, southeastern Cameroon.

|  |  |  |  |  |  |  |  |  |  |
| --- | --- | --- | --- | --- | --- | --- | --- | --- | --- |
|  |  | Dwellings | |  | Inhabitants per Household | | | |  |
| Village | Location | Total | Occupied |  | Mean | SD | Min | Max | Total Inhabitants |
| Abing- Nkolemboula | N 02°42'20''/E013°19'43'' | 14 | 12 |  | 4.92 | 3.75 | 1 | 13 | 59 |
| Adjap-Mintom | N 02°40'16"/E 013°15'13" | 20 | 15 |  | 3.6 | 2.32 | 1 | 8 | 54 |
| Akom | N 02°37'28''/E 013°19'11'' | 37 | 24 |  | 4.63 | 3.16 | 1 | 17 | 93 |
| Akonetyé | N 02°42'03''/E 013°00'08'' | 32 | 23 |  | 5.05 | 2.5 | 1 | 11 | 111 |
| Assok | N 02°39'21''/E 013°17'12'' | 36 | 23 |  | 3.3 | 2.87 | 1 | 12 | 106 |
| Belle Ville | N 02°35'23''/E 013°21'48'' | 11 | 10 |  | 4.1 | 2.18 | 1 | 8 | 76 |
| Bemba II | N 02°44'44,5''/ E 013°21'13'' | 20 | 14 |  | 4.43 | 2.38 | 1 | 8 | 41 |
| Doum | N 02°40'16"/ E 013°15'13" | 30 | 24 |  | 4.54 | 2.41 | 1 | 13 | 62 |
| Meyos-Mintom | N 02°43'30''/E 013°20'50'' | 7 | 5 |  | 5 | 3.32 | 2 | 9 | 109 |
| Odoumou | E 02°39'32''/E 013°39'20'' | 30 | 22 |  | 4.23 | 2.93 | 1 | 11 | 25 |
| Grand Total |  | 237 | 172 |  | 4.33 | 2.77 | 1 | 17 | 736 |
|  |  |  |  |  |  |  |  |  |  |

Supplementary Table 2. Description of diversity indices used for the communities of hunted animals by village.

1. Species richness, i.e. the total number of species recorded into each habitat type;
2. Dominance (D):
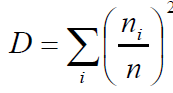
with values ranging from 0 to 1, with 0 when all taxa are equally present and 1 when only one taxon is found in the sample. In this formula, n*_i_* is the number of individuals of taxon *i* and n is the total sample size.
3. Simpson index: *S* = 1 ̶ *D*.
4. Shannon-Wiener index (Shannon & Weaver, 1963): *H*’ = − Σ[*n*/*N* log (*n*/*N*)] where n is the number of individuals of each species in each village and *N* is the total number of individuals of all taxa that were recorded in each village.
5. Evenness, calculated by Pielou’s formula: *e* = *H*’/log *S* with *H*’ representing Shannon’s index, and *S* the total number of bird species observed in each habitat type (Magurran, 1988).
6. Chao 1, that is the number of species predicted to be present at each study area given the sample observed (Hughes et al. 2001; Chodak et al. 2013): Chao1 = *S* + *F*_1_ (*F*_1_ – 1) / [2 (*F*_2_ + 1)] where S is the species richness, F_1_ is the number of singletons and F_2_ is the number of doubletons in each village.

Supplementary Table 3. Raw data

Excel sheet **Supplementary_Table3.xlsx**

Supplementary Table 4.. List of hunted species, IUCN Red List status and Cameroon law Class, and numbers of carcasses recorded for each village in the ten study villages in southeastern Cameroon.

Excel sheet **Supplementary_Table4.xlsx**

Supplementary Table 5. Diversity metrics (with upper and lower 95% confidence intervals, generated after 10,000 random Monte Carlo permutations) for the communities of hunted animals by village.

1. Prey groups

|  | Taxa_S | Dominance_D | Simpson_1-D | Shannon_H | Evenness_e^H/S | Margalef | Chao-1 |
| --- | --- | --- | --- | --- | --- | --- | --- |
| **Adjap** | 8 | 0.2703 | 0.7297 | 1.574 | 0.6031 | 1.548 | 8 |
| Lower | 8 | 0.225 | 0.6593 | 1.358 | 0.4893 | 1.548 | 8 |
| Upper | 8 | 0.3405 | 0.7748 | 1.713 | 0.6935 | 1.548 | 9 |
| **Akom** | 7 | 0.2513 | 0.7487 | 1.579 | 0.6926 | 1.013 | 7 |
| Lower | 7 | 0.2305 | 0.7201 | 1.493 | 0.6356 | 1.013 | 7 |
| Upper | 7 | 0.2799 | 0.7694 | 1.643 | 0.7385 | 1.013 | 7 |
| **Akonetyé** | 6 | 0.6799 | 0.3201 | 0.6376 | 0.3153 | 0.9864 | 9 |
| Lower | 6 | 0.5952 | 0.2363 | 0.5162 | 0.2793 | 0.9864 | 6 |
| Upper | 6 | 0.7637 | 0.4048 | 0.799 | 0.3705 | 0.9864 | 9 |
| **Assok** | 4 | 0.4491 | 0.5509 | 0.905 | 0.618 | 0.6667 | 4 |
| Lower | 4 | 0.4101 | 0.5099 | 0.7969 | 0.5547 | 0.6667 | 4 |
| Upper | 4 | 0.4901 | 0.5899 | 1.019 | 0.6927 | 0.6667 | 5 |
| **Belle-Ville** | 7 | 0.3805 | 0.6195 | 1.246 | 0.4965 | 1.223 | 7 |
| Lower | 6 | 0.3262 | 0.5463 | 1.069 | 0.4203 | 1.019 | 6 |
| Upper | 7 | 0.4536 | 0.6736 | 1.38 | 0.5706 | 1.223 | 8 |
| **Bemba** | 7 | 0.2763 | 0.7237 | 1.488 | 0.6327 | 1.048 | 7 |
| Lower | 7 | 0.2513 | 0.691 | 1.398 | 0.578 | 1.048 | 7 |
| Upper | 7 | 0.3088 | 0.7486 | 1.563 | 0.6816 | 1.048 | 7 |
| **Doum** | 8 | 0.2677 | 0.7323 | 1.601 | 0.6201 | 1.176 | 8 |
| Lower | 8 | 0.2381 | 0.6954 | 1.509 | 0.5655 | 1.176 | 8 |
| Upper | 8 | 0.3045 | 0.7618 | 1.678 | 0.6694 | 1.176 | 8 |
| **Meyos** | 7 | 0.2277 | 0.7723 | 1.706 | 0.7868 | 1.121 | 7 |
| Lower | 7 | 0.1971 | 0.7265 | 1.59 | 0.7006 | 1.121 | 7 |
| Upper | 7 | 0.2734 | 0.8029 | 1.779 | 0.8466 | 1.121 | 7 |
| **Nkolemboula** | 7 | 0.2152 | 0.7848 | 1.699 | 0.7812 | 1.072 | 7 |
| Lower | 7 | 0.1964 | 0.756 | 1.604 | 0.7101 | 1.072 | 7 |
| Upper | 7 | 0.2439 | 0.8036 | 1.761 | 0.8314 | 1.072 | 7 |
| **Odoumou** | 7 | 0.3049 | 0.6951 | 1.49 | 0.6338 | 1.207 | 7 |
| Lower | 7 | 0.2532 | 0.6239 | 1.319 | 0.5351 | 1.207 | 7 |
| Upper | 7 | 0.3759 | 0.7468 | 1.608 | 0.7131 | 1.207 | 7 |
|  |  |  |  |  |  |  |  |

1. Prey species

|  |  |  |  |  |  |  |  |
| --- | --- | --- | --- | --- | --- | --- | --- |
|  | Taxa_S | Dominance_D | Simpson_1-D | Shannon_H | Evenness_e^H/S | Margalef | Chao-1 |
| **Adjap** | 17 | 0.1421 | 1 | 2.298 | 1 | 3.497 | 17.43 |
| Lower | 16 | 0.1126 | 1 | 2.119 | 0 | 3.279 | 17 |
| Upper | 17 | 0.1812 | 1 | 2.451 | 1 | 3.497 | 27.5 |
| **Akom** | 31 | 0.1054 | 1 | 2.663 | 0.4626 | 5.037 | 38 |
| Lower | 31 | 0.09202 | 1 | 2.564 | 0.4195 | 5.037 | 31.38 |
| Upper | 31 | 0.1219 | 1 | 2.766 | 0.513 | 5.037 | 49 |
| **Akonetyé** | 13 | 0.2271 | 1 | 1.767 | 0 | 2.359 | 18 |
| Lower | 12 | 0.1955 | 1 | 1.64 | 0 | 2.162 | 12 |
| Upper | 13 | 0.2657 | 1 | 1.904 | 1 | 2.359 | 23 |
| **Assok** | 11 | 0.1829 | 0.8171 | 1.9 | 1 | 2.201 | 14 |
| Lower | 10 | 0.1541 | 1 | 1.767 | 1 | 1.981 | 10 |
| Upper | 11 | 0.2232 | 1 | 2.037 | 1 | 2.201 | 20 |
| **Belle-Ville** | 20 | 0.1621 | 1 | 2.27 | 0 | 3.879 | 24.2 |
| Lower | 17 | 0.1269 | 1 | 2.083 | 0 | 3.267 | 17.75 |
| Upper | 20 | 0.2071 | 1 | 2.433 | 1 | 3.879 | 38 |
| **Bemba** | 27 | 0.1656 | 0.8344 | 2.335 | 0.3825 | 4.558 | 29.14 |
| Lower | 26 | 0.1389 | 1 | 2.21 | 0 | 4.383 | 26.86 |
| Upper | 27 | 0.1917 | 1 | 2.474 | 0.4423 | 4.558 | 42 |
| **Doum** | 38 | 0.1004 | 1 | 2.875 | 0 | 6.226 | 42.67 |
| Lower | 37 | 0.08349 | 1 | 2.751 | 0.4133 | 6.058 | 38.46 |
| Upper | 38 | 0.1221 | 1 | 2.977 | 1 | 6.226 | 53 |
| **Meyos** | 38 | 0.05155 | 1 | 3.254 | 1 | 6.872 | 43.6 |
| Lower | 37 | 0.04524 | 1 | 3.132 | 0.6053 | 6.686 | 38.38 |
| Upper | 38 | 0.0643 | 1 | 3.325 | 1 | 6.872 | 53 |
| **Nkolemboula** | 35 | 0.08232 | 1 | 2.943 | 1 | 6.093 | 36.67 |
| Lower | 35 | 0.06825 | 1 | 2.821 | 0 | 6.093 | 35.38 |
| Upper | 35 | 0.09895 | 1 | 3.054 | 1 | 6.093 | 50 |
| **Odoumou** | 31 | 0.09586 | 1 | 2.779 | 1 | 5.911 | 37.88 |
| Lower | 27 | 0.07852 | 1 | 2.597 | 0 | 5.123 | 29.6 |
| Upper | 31 | 0.122 | 1 | 2.91 | 1 | 5.911 | 55 |
|  |  |  |  |  |  |  |  |

**Supplementary References**

Hughes, J. B., Hellmann, J. J., Ricketts, T. H., & Bohannan, B. J. (2001). Counting the uncountable: statistical approaches to estimating microbial diversity. Appl. Environ. Microbiol., 67(10), 4399-4406.

Chodak, M., Gołębiewski, M., Morawska-Płoskonka, J., Kuduk, K., & Niklińska, M. (2013). Diversity of microorganisms from forest soils differently polluted with heavy metals. Applied Soil Ecology, 64, 7-14.

IUCN. 2020. The IUCN Red List of Threatened Species v. 2015.2. Available at <https://www.iucnredlist.org>

Republic of Cameroon. 1994. Loi No 94/01 du 20 janvier 1994 portant régime des forêts, de la faune et de la pêche.
